# 30-year Analysis of Designated International Safe Communities

**Published:** 2019-08

**Authors:** Dale Hanson

**Affiliations:** ^*a*^ Associate Professor, Chair. International Safe Community Certifying Center, Australia

## Abstract

**Background:**

The International Safe Communities movement was established in 1986, and since that time has developed into an international collaborative network comprised of the WHO CCCSP/ ISCCC, regional and national safe community organizations, support centers and communities. Purpose to review the outcomes of communities who have achieved designation as an international safe community over the 32-year period from 1986 to 2018.

**Methods:**

An audit of the ISCCC database and website was undertaken. Any inconsistencies were checked for accuracy against the original designation application.

**Results:**

Since the safe community movement began in 1998 four hundred and three International Safe Communities have been designated. Data was available for analysis regarding 361 communities. Thirty-eight communities, all designated prior to 2006, had been removed from the data base prior to this data being handed over to the ISCCC. The data set is complete from 2006 onwards. The program has a high reach, 86 million people have been served by a safe community program that has achieved International Safe Community Designation on at least one occasion (1.1% of the world population). Forty-nine millions of these are served by safe community programs whose certification remains active. In 2018 there were 39 certifications (13 designations and 26 redesignations), covering a population of 7.1 million people. One hundred and sixty-one (40%) of the original 403 communities have maintained a safe community certification. Two hundred and forty-four (68%) communities only sought initial designation, 92 (25%) were designated twice, 22 (6%) were designated three times and 3 (1%) were designated four times. International Safe Communities vary considerably in size from the smallest with a population of 1,000 inhabitants to the largest of 12 million. However, the distribution is skewed towards smaller communities (5th percentile = 6,000, a median of 90,000, a mean of 240,000 and a 95th percentile of 700,000.

**Conclusions:**

The International Safe Community movement has accomplished remarkable growth in the 30 years since it began in Sweden in the late 1980s. The program has achieved high reach. Four hundred and three communities have been designated by the end of 2018 with a population footprint of 86 million people.

**Keywords:**

International Safe Communities, Designation, Sustainability, Evaluation

